# *Brucella* prosthetic joint infection following total knee arthroplasty: a rare case report

**DOI:** 10.3389/fsurg.2026.1853630

**Published:** 2026-09-21

**Authors:** Faten Almohideb, Abdulmonem Alkhateeb, Mohammad Alboukai, Yousef Aldobikhi, Kadi AlOtaibi, Ghada Alnowaiser, Abdullah Alfaqih

**Affiliations:** 1 Department of Radiology and Medical Imaging, College of Medicine, King Saud University, King Saud University Medical City, Riyadh, Saudi Arabia; 2 Radiology Department, King Faisal General Hospital, Al Ahsa Health Cluster, Al Ahsa, Eastern Province, Saudi Arabia; 3 College of Medicine, Princess Nourah Bint Abdulrahman University, Riyadh, Saudi Arabia

**Keywords:** *Brucella*, case report, periprosthetic infection, prosthetic joint infection, total knee arthroplasty

## Abstract

**Background:**

Brucellosis is a zoonotic infection with a high prevalence in endemic regions and a strong predilection for osteoarticular involvement. *Brucella* prosthetic joint infection (PJI) is an exceptionally rare but challenging complication of total knee arthroplasty (TKA), often characterized by delayed presentation, indolent symptoms, and diagnostic difficulty. There is no consensus regarding optimal management, particularly concerning surgical strategy and duration of antimicrobial therapy.

**Methods:**

A narrative literature review was conducted using PubMed and Scopus, searched on 1 March 2026 to identify reported cases of *Brucella* PJI following TKA. Cases were included based on microbiological or serological confirmation of *Brucella* infection. The screening process, exclusion criteria, and handling of duplicate reports are described in the Methods, and the selection process is summarized in a flow diagram.

**Case presentation:**

We report the case of a 67-year-old male farmer who keeps sheep and presented four years after bilateral TKA with a three-week history of progressive pain and swelling of the right knee, without systemic symptoms. Laboratory evaluation revealed a markedly elevated C-reactive protein with a normal leukocyte count. Ultrasound-guided joint aspiration and subsequent intraoperative cultures confirmed *Brucella* species infection, supported by a positive Rose Bengal test and a standard agglutination titre above 1:160. Imaging demonstrated loosening of the tibial component with scintigraphic findings suggestive of PJI. The patient was treated with prolonged anti-brucellosis therapy followed by first-stage revision arthroplasty, including complete implant removal, extensive debridement, and insertion of an antibiotic-loaded articulating cement spacer. Postoperatively, he received combination doxycycline and rifampin therapy with an uncomplicated clinical course. *Brucella* was still recovered from the intraoperative cultures despite a completed three-month preoperative course, and a second three-month course was given. Second-stage reimplantation was performed on November 19, 2025, with negative intraoperative cultures. At the most recent review on May 11, 2026, approximately six months after reimplantation, he was pain free with a range of motion of 0° to 120°, a normal C-reactive protein, and no evidence of recurrence.

**Results:**

Forty cases of *Brucella* PJI after TKA were identified. Most presented years after index surgery with non-specific symptoms. Management strategies varied, with implant stability guiding the choice between medical therapy alone and surgical revision, most commonly a two-stage approach.

**Conclusion:**

*Brucella* PJI should be considered in painful arthroplasties in endemic settings. Successful management requires a high index of suspicion, appropriate microbiological diagnosis, prolonged combination antimicrobial therapy, and individualized surgical intervention based on implant stability. Persistence of *Brucella* in intraoperative cultures despite three months of appropriate systemic therapy, as seen here, argues that implant retention is inadequate once components are radiographically loose.

## 1 Introduction

Brucellosis is a globally distributed zoonotic infection caused by various species of the Gram-negative coccobacilli *Brucella* (1). Primarily transmitted to humans through the ingestion of unpasteurized dairy products, direct contact with infected livestock, or the inhalation of contaminated aerosols, it remains highly endemic in the Middle East, the Mediterranean basin, Central Asia, and parts of Latin America (2). While the disease often presents as a systemic febrile illness, classically known as “undulant fever”, its most frequent focal complication is osteoarticular involvement, occurring in 2%–85% of cases. Common manifestations include spondylodiscitis, sacroiliitis, and peripheral arthritis of large weight-bearing joints (3).

In the field of orthopedic surgery, total knee arthroplasty (TKA) has become a highly successful intervention for end-stage joint destruction. However, periprosthetic joint infection (PJI) remains a devastating complication, leading to significant patient morbidity and a substantial economic burden on healthcare systems (4). While the majority of these infections are caused by aerobic Gram-positive cocci such as Staphylococcus aureus and coagulase-negative staphylococci, PJI due to *Brucella* species is exceedingly rare. Since the first documented case in 1991, approximately 30–40 cases of *Brucella* prosthetic knee infections have been reported in the English medical literature (5).

Diagnosing *Brucella* PJI poses a significant clinical challenge due to its indolent and subacute clinical course (6). Patients often present years after the initial surgery with non-specific symptoms such as progressive pain and swelling, sometimes in the absence of systemic markers like fever (7). *Brucella* is also a fastidious, slow-growing organism that often fails to grow in standard 48-h cultures. Standard synovial fluid cultures are only positive in roughly 75% of cases, and blood cultures yield positive results in only 30% (8). Consequently, many cases are initially misdiagnosed as aseptic loosening or culture-negative PJI. Accurate diagnosis necessitates a high index of clinical suspicion combined with specialized laboratory techniques, including serological tests (e.g., Rose Bengal, tube agglutination tests) and prolonged incubation of intraoperative tissue samples for up to 21 days (9).

There is currently no established gold standard for the management of *Brucella* PJI. Treatment strategies generally depend on the radiographic stability of the implant. Emerging evidence suggests that in the absence of component loosening, prolonged combination antibiotic therapy, typically involving doxycycline and rifampicin with or without an aminoglycoside, can result in successful infection eradication and implant retention (5). However, if radiographic loosening is present, surgical revision is mandatory. While two-stage revision has been the most commonly performed surgical procedure, recent reports have also demonstrated favorable outcomes with one-stage revision in selected patients (10).

Given the pathogen's ability to persist intracellularly and cause late reactivation, clinicians must remain vigilant when treating painful prostheses in endemic regions or in patients with relevant travel and dietary histories. Because of the high risk of nosocomial transmission through aerosolization, healthcare and laboratory personnel must utilize specialized personal protective equipment, including FFP3 respirators, when handling suspected *Brucella* clinical samples or performing invasive joint procedures (11).

## 2 Materials and methods

### 2.1 Case presentation

A 67-year-old male with no known comorbidities underwent bilateral TKA in March 2021. His postoperative course was uneventful, achieving a full range of motion and satisfactory functional outcomes. He is retired but continues to work as a farmer and keeps sheep on his own farm, representing both occupational and direct animal-contact exposure to *Brucella* species. He denied consumption of unpasteurized dairy products and reported no recent travel. Progressive pain and swelling of the right knee began at approximately March 20, 2025. On April 10, 2025, he presented with a 3-week history of these symptoms, which had worsened over that interval. He was afebrile and reported no weight loss, night sweats, or other constitutional symptoms. The full clinical course is summarized in Table 1.

**Table 1:** Timeline of the clinical course, reported according to the CARE guidelines.

Date	Event
9 March 2021	Index bilateral total knee arthroplasty
≈20 March 2025	Symptom onset. Progressive right knee pain and swelling began approximately three weeks before hospital presentation and worsened over that period
10 April 2025	First presentation, with a three-week history of progressive right knee pain and swelling. Examination: localized warmth and tenderness, mild swelling, joint effusion; no erythema; scar clean and well healed with no sinus tract; range of motion 5–120°; afebrile (36.6–37.2 °C); hemodynamically stable. Same-day ultrasound-guided aspiration: approximately 20 mL of yellow, cloudy, turbid fluid; leukocytes 10,000 cells/µL with >69% neutrophils. Laboratory: CRP 119.08 mg/L, leukocytes 3.98 × 10^9^/L
12 April 2025	Synovial fluid Gram stain negative on days 1 and 2
13 April 2025	Blood cultures, fungal smear and culture, and acid-fast bacilli smear and culture obtained. Blood cultures negative; no fungal elements and no acid-fast bacilli on smear
19 April 2025	Synovial fluid culture positive after seven days of incubation: Gram-negative coccobacilli consistent with *Brucella* species. Rose Bengal test positive; standard tube agglutination titre >1:160
19 April 2025	Preoperative therapy started: doxycycline 100 mg orally twice daily and rifampin 600 mg orally once daily for three months, under Infectious Diseases supervision
6 May 2025	Bone scintigraphy: intense hyperemia and increased delayed uptake around the right knee prosthesis
11 May 2025	Gallium scintigraphy: incongruent focal uptake around the tibial and femoral components, suggestive of prosthetic joint infection
25 May 2025	Fungal culture final report: no growth at six weeks
12 June 2025	Mycobacterial culture final report: no growth at eight weeks
14 July 2025	Admission for first-stage revision. Laboratory on the day of admission: CRP 16.28 mg/L, leukocytes 5.07 × 10^9^/L. Radiograph: radiolucency beneath the tibial component, consistent with loosening
16 July 2025	First-stage revision: removal of all components and cement, radical synovectomy, extensive debridement, and placement of a vancomycin-loaded articulating polymethylmethacrylate spacer. Intra-operative synovial fluid: leukocytes 8,000 cells/µL with >67% neutrophils
18 July 2025	Intra-operative Gram stain negative on days 1 and 2. Blood, fungal, and mycobacterial cultures negative
25 July 2025	Intra-operative culture positive after seven days of incubation: Gram-negative coccobacilli consistent with *Brucella* species
25 July 2025	Second course of doxycycline 100 mg orally twice daily and rifampin 600 mg orally once daily started, for three months
27 July 2025	Discharge: stable, afebrile, wound clean and dry, on doxycycline and rifampin
15 August 2025	Follow-up: wound completely healed, clips removed, range of motion 10–90°, mobilizing with assistance. Radiograph: cement spacer *in situ*
11 October 2025	Follow-up: stable on the spacer, no pain, no signs of recurrent infection. Booked for second-stage reimplantation
19 October 2025	Laboratory: leukocytes 3.5 × 10^9^/L, erythrocyte sedimentation rate 22 mm/h, CRP 4.9 mg/L
19 November 2025	Second-stage reimplantation with revision components. Intra-operative laboratory studies and cultures, including for *Brucella*, all negative. No further antimicrobial therapy given
20 November 2025	Postoperative radiograph: revision components *in situ*, no acute hardware-related complication and no loosening
23 November 2025	Discharge: stable, afebrile, wound clean and dry
19 February 2026	Follow-up, three months after reimplantation: clinically well. CRP 14.15 mg/L, attributed to postoperative inflammation
11 May 2026	Most recent follow-up, approximately six months after reimplantation: no complaints, ambulating without an assistive device, wound healed with no sinus or discharge, range of motion 0–120°, good quadriceps strength and patellar tracking. CRP 3.2 mg/L. Radiographs: no loosening or hardware-related complication. Gallium scan negative

On examination at the first hospital visit on April 10, 2025, the right knee showed localized warmth and tenderness with mild swelling and a joint effusion. There was no erythema and no wound complication. The surgical scar was clean and well healed, with no sinus tract. Range of motion was 5°–120°. Recorded temperatures were 36.6 °C–37.2 °C, and no fever was documented at any point during the illness. He was hemodynamically stable and appeared generally well, with mild pain and discomfort attributable to the right knee.

On the day of presentation, ultrasound-guided joint aspiration was performed by musculoskeletal interventional radiology (Figure 1). Approximately 20 mL of yellow, cloudy, turbid fluid was aspirated. The synovial leukocyte count was 10,000 cells/µL with a granulocyte predominance above 69%. Gram stain was negative on days 1 and 2, and Gram-negative coccobacilli grew after seven days of incubation. Culture of the aspirate identified *Brucella* species. Laboratory investigations demonstrated a markedly elevated C-reactive protein (CRP) level of 119.08 mg/L, with a leukocyte count of 3.98 × 10^9^/L. The Rose Bengal test was positive and the standard tube agglutination (Wright) test titre exceeded 1:160, which is diagnostic of active infection. Blood cultures obtained on April 13, 2025, were negative, as were fungal and mycobacterial smears and cultures, with no growth at six and eight weeks respectively. No other organism was isolated. The complete diagnostic work-up for both sampling episodes is summarized in Table 2. Plain radiographs obtained on July 14, 2025, on the day of admission for revision surgery, showed radiolucency beneath the right tibial component, consistent with implant loosening (Figure 2). A bone scintigraphy performed on May 6, 2025, revealed intense hyperemia and increased delayed uptake surrounding the right knee prosthesis (Figure 3). Subsequent gallium scintigraphy on May 11, 2025, demonstrated incongruent focal uptake around both the tibial and femoral components, raising suspicion for prosthetic joint infection.

**Figure 1:**
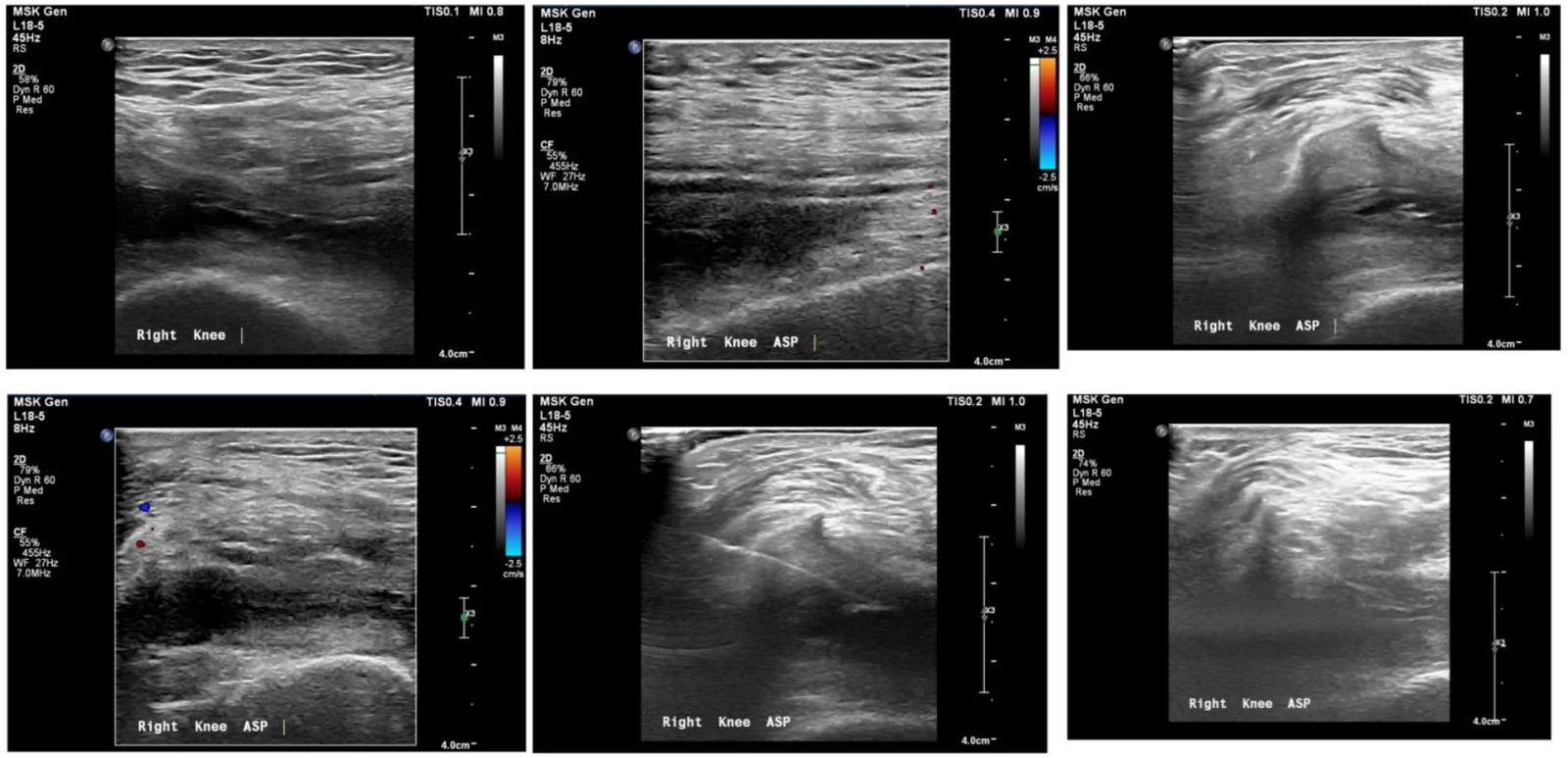
Ultrasound examination of the right knee demonstrating peri-prosthetic joint effusion. Ultrasound-guided aspiration was performed by musculoskeletal interventional radiology, yielding synovial fluid that subsequently grew *Brucella* species.

**Table 2:** Diagnostic work-up at the two sampling episodes. No organism other than *Brucella* species was isolated.

Parameter	Diagnostic aspiration, 10 April 2025	Intra-operative sampling, 16 July 2025
Macroscopic appearance	Approximately 20 mL of yellow, cloudy, turbid fluid	Not applicable
Synovial leukocyte count	10,000 cells/µL	8,000 cells/µL
Differential	Granulocyte predominance, >69%	Granulocyte predominance, >67%
Gram stain	Negative on days 1 and 2 (12 April 2025)	Negative on days 1 and 2 (18 July 2025)
Culture result	Gram-negative coccobacilli after seven days (19 April 2025)	Gram-negative coccobacilli after seven days (25 July 2025)
Incubation duration	Seven days	Seven days
Species-level identification	Not performed	Not performed
Rose Bengal test	Positive	Positive
Standard tube agglutination (Wright) test	Titre >1:160, diagnostic of active infection	Titre >1:160
Blood cultures	Negative (13 April 2025)	Negative (18 July 2025)
Fungal smear and culture	No fungal elements on smear (13 April 2025); no growth at six weeks (final report 25 May 2025)	Negative (18 July 2025)
Mycobacterial smear and culture	No acid-fast bacilli on smear (13 April 2025); no growth at eight weeks (final report 12 June 2025)	Negative (18 July 2025)

**Figure 2:**
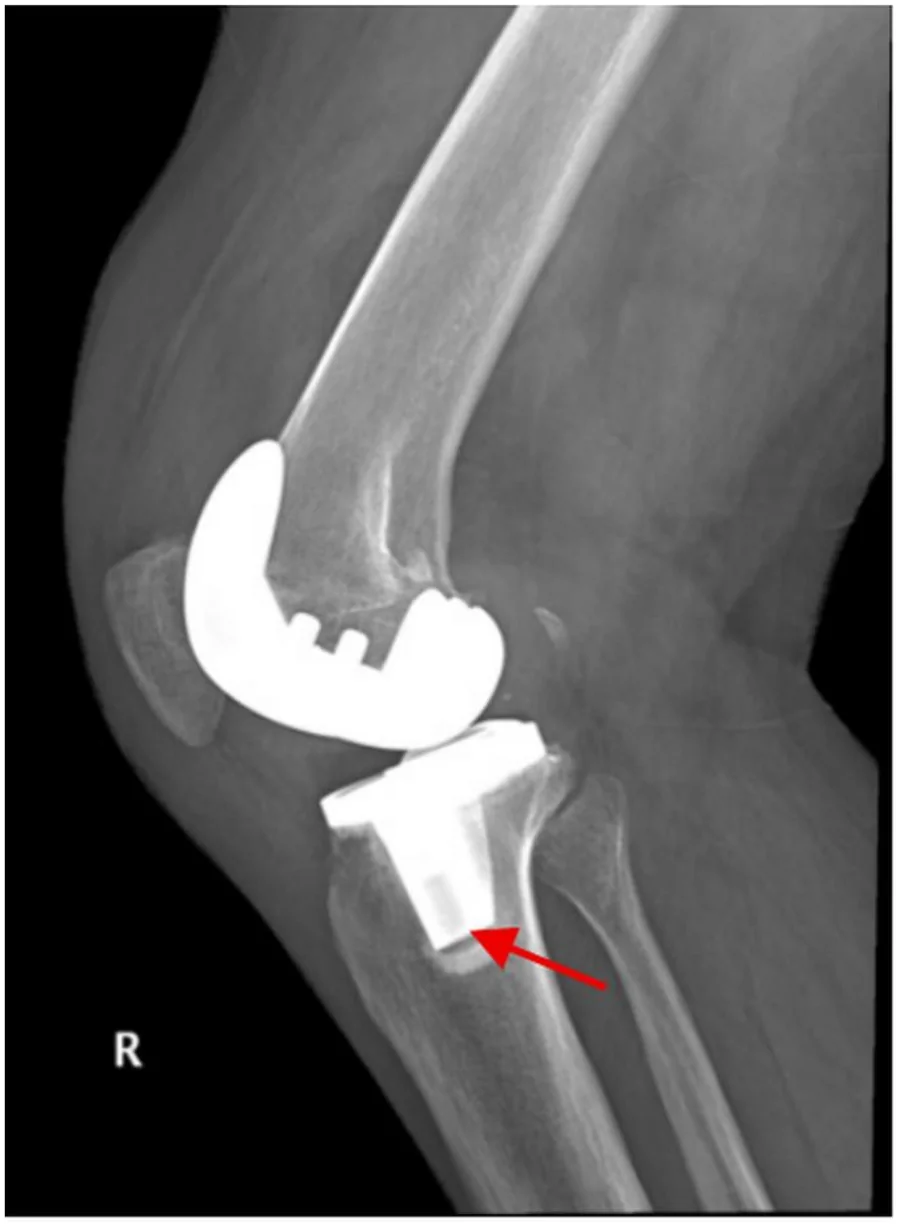
Lateral radiograph of the right knee obtained on 14 July 2025, before first-stage revision. There is a large joint effusion, with a small focal area of lucency at the distal tip of the tibial component (red arrow), suggestive of loosening.

**Figure 3:**
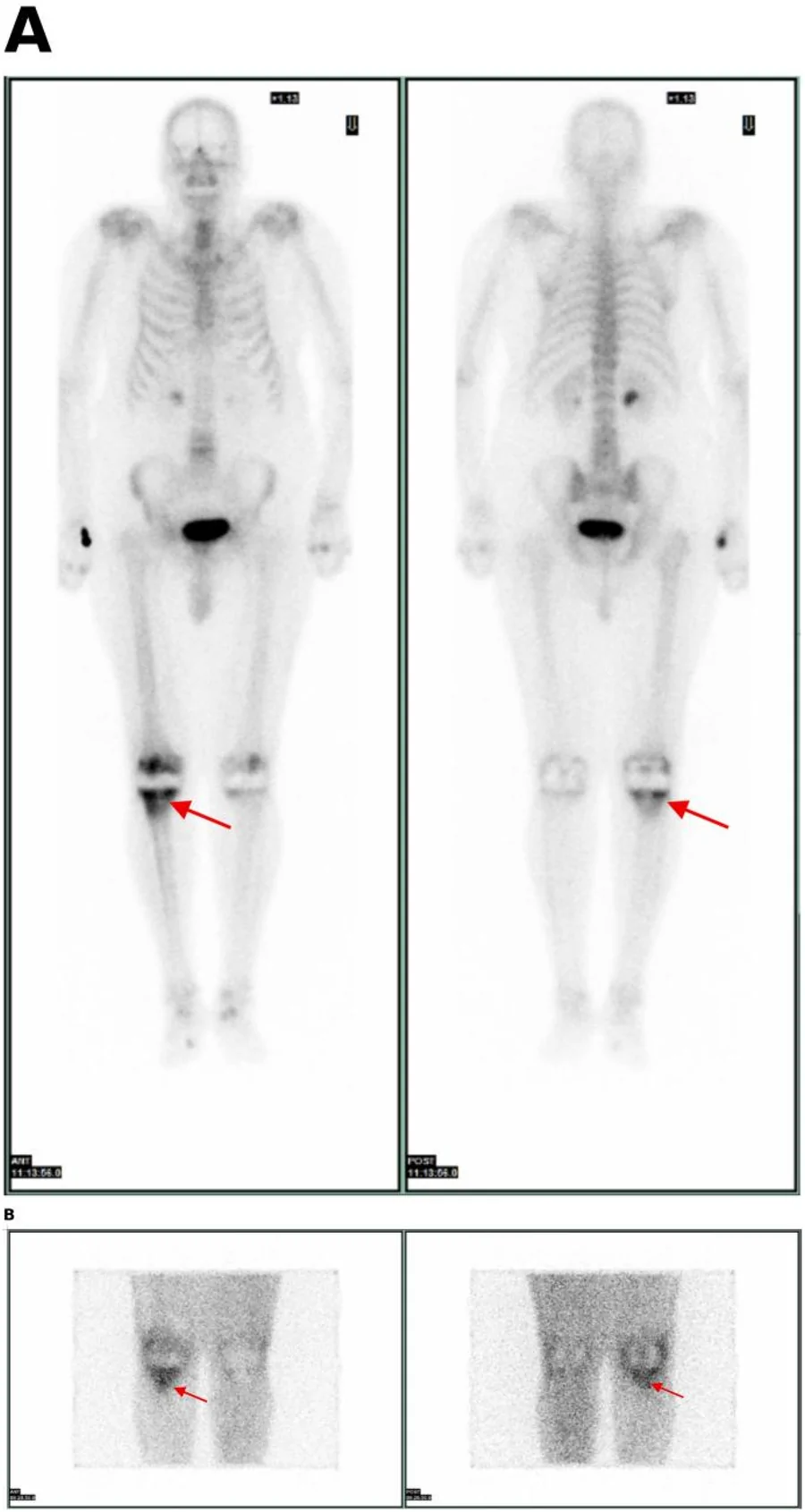
Nuclear medicine imaging of the right knee. **(A)** Whole-body bone scintigraphy on 6 May 2025, anterior and posterior views, showing intense hyperemia and delayed uptake around the right knee prosthesis (red arrows), concerning for infection. **(B)** Gallium scintigraphy on 11 May 2025, anterior and posterior views, showing incongruent moderate focal uptake surrounding the right knee tibial prosthesis, together with a small focus of mild incongruent uptake at the lateral aspect of the femoral prosthesis (red arrows), suggestive of active infection.

Anti-brucellosis therapy was started on April 19, 2025, under Infectious Diseases supervision, with oral doxycycline 100 mg twice daily and rifampin 600 mg once daily for three months. *Brucella* is a facultative intracellular organism, and at least three months of combination therapy is recommended for focal osteoarticular disease because shorter regimens carry a high relapse rate (35). The preoperative course was intended to control active infection and reduce the bacterial burden before the planned exchange. The patient completed the course in full, and CRP fell from 119.08 mg/L at presentation to 16.28 mg/L on July 14, 2025, the day of admission for surgery. On July 16, 2025, he underwent first-stage revision arthroplasty. Intra-operatively, loosening of the tibial component was noted, without evidence of gross abscess formation or osteomyelitis. All prosthetic components and cement were removed, followed by radical synovectomy and extensive debridement. An antibiotic-loaded articulating cement spacer using vancomycin-impregnated polymethylmethacrylate was implanted. Vancomycin has no activity against *Brucella*, and the local antibiotic was not intended to treat the *Brucella* infection. It was directed at Gram-positive organisms, which are the commonest cause of prosthetic joint infection and a recognized risk for superimposed contamination during a staged exchange, while the articulating spacer itself maintained the joint space, soft tissue tension, and knee motion between stages. Eradication of *Brucella* relied on systemic doxycycline and rifampin, which achieve the intracellular concentrations this organism requires and that local elution from cement cannot provide. Intra-operative synovial fluid showed a leukocyte count of 8,000 cells/µL with a granulocyte predominance above 67%, and cultures again grew *Brucella* species after seven days of incubation, despite the completed three-month preoperative course. This persistence confirmed that systemic therapy alone would not eradicate biofilm-associated infection of the prosthesis and supported proceeding with implant removal rather than retention.

Postoperatively, in collaboration with the Infectious Disease team, the patient was started on oral doxycycline (100 mg twice daily) and rifampin (600 mg once daily) on July 25, 2025, for a planned duration of three months. The Infectious Diseases service recommended three months of doxycycline and rifampin before surgery, a second three-month course of the same regimen after the first-stage revision, and reimplantation only once the full antibiotic course was complete, inflammatory markers had normalized, and there was no clinical evidence of persistent infection. The patient was compliant with both courses, and no adverse drug reaction was recorded. He received standard deep vein thrombosis prophylaxis and was maintained on partial weight-bearing with supervised physiotherapy. The postoperative course was uncomplicated, and the patient remained afebrile and clinically stable throughout hospitalization.

The patient was discharged on July 27, 2025, in stable condition, with a clean and dry surgical wound and continued anti-brucellosis therapy with doxycycline and rifampin. At follow-up on August 15, 2025, the wound had completely healed, surgical clips were removed, and the patient demonstrated a knee range of motion from 10° to 90° while mobilizing with assistance (Figure 4). At review on October 11, 2025, he remained clinically stable on the cement spacer, with no pain or signs of recurrent infection. Inflammatory markers on October 19, 2025, were within normal limits, with a leukocyte count of 3.5 × 10^9^/L, an erythrocyte sedimentation rate of 22 mm/h, and a CRP of 4.9 mg/L, and second-stage reimplantation was scheduled. Second-stage reimplantation with revision components was performed on November 19, 2025, after completion of the full antibiotic course. Intra-operative samples were sent for the same laboratory studies and cultures as at the first stage, and all were negative, including for *Brucella*. No further antimicrobial therapy was given. Plain radiographs obtained on November 20, 2025, showed revision components *in situ* with expected postoperative changes and no evidence of acute hardware-related complication or loosening (Figure 5). He was discharged on November 23, 2025, stable, afebrile, with a clean and dry wound.

**Figure 4:**
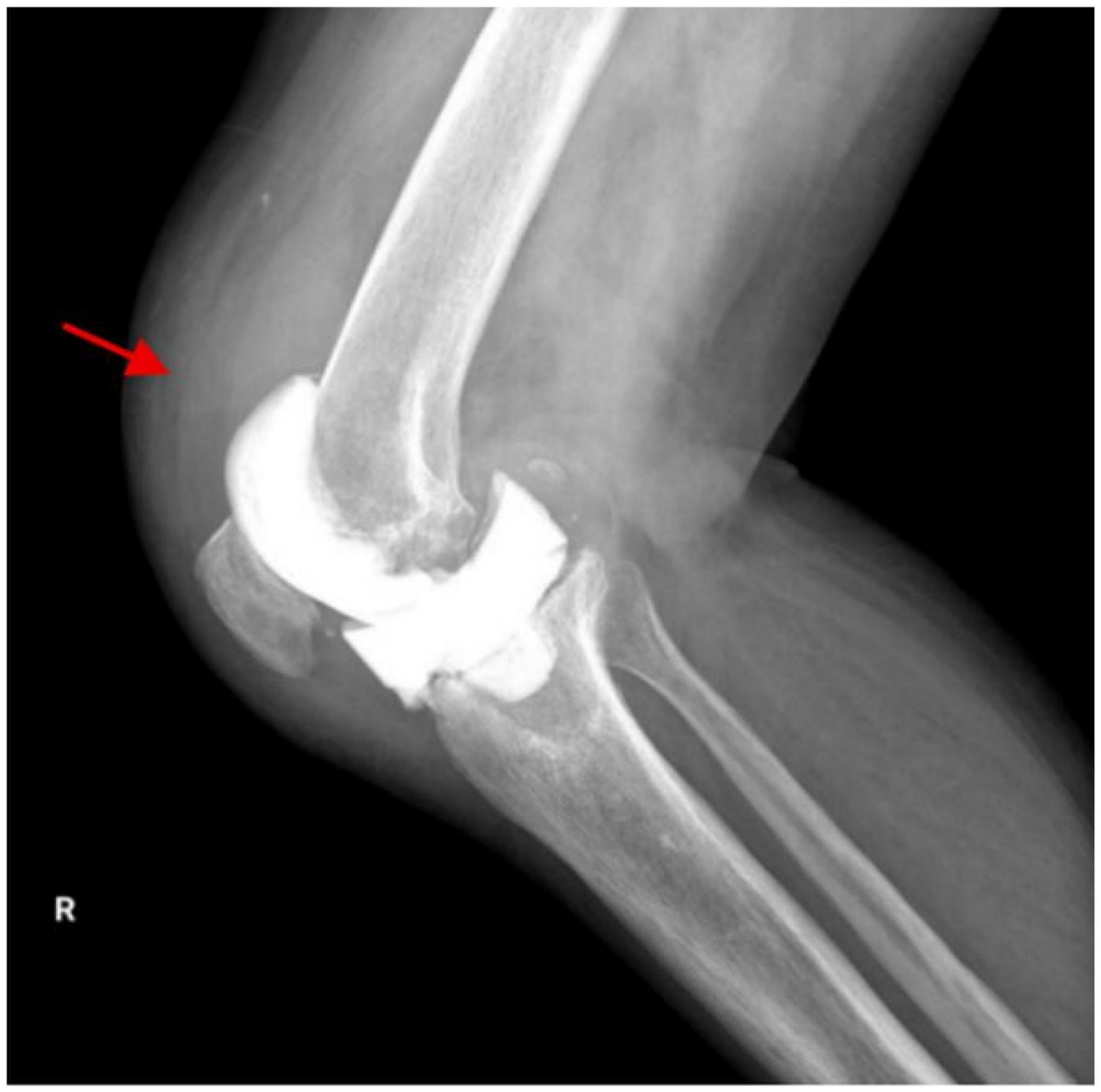
Lateral radiograph of the right knee obtained on 15 August 2025, after first-stage revision of the right knee by placement of an antibiotic-loaded articulating cement spacer (vancomycin-impregnated polymethylmethacrylate). A large joint effusion is still seen (red arrow).

**Figure 5:**
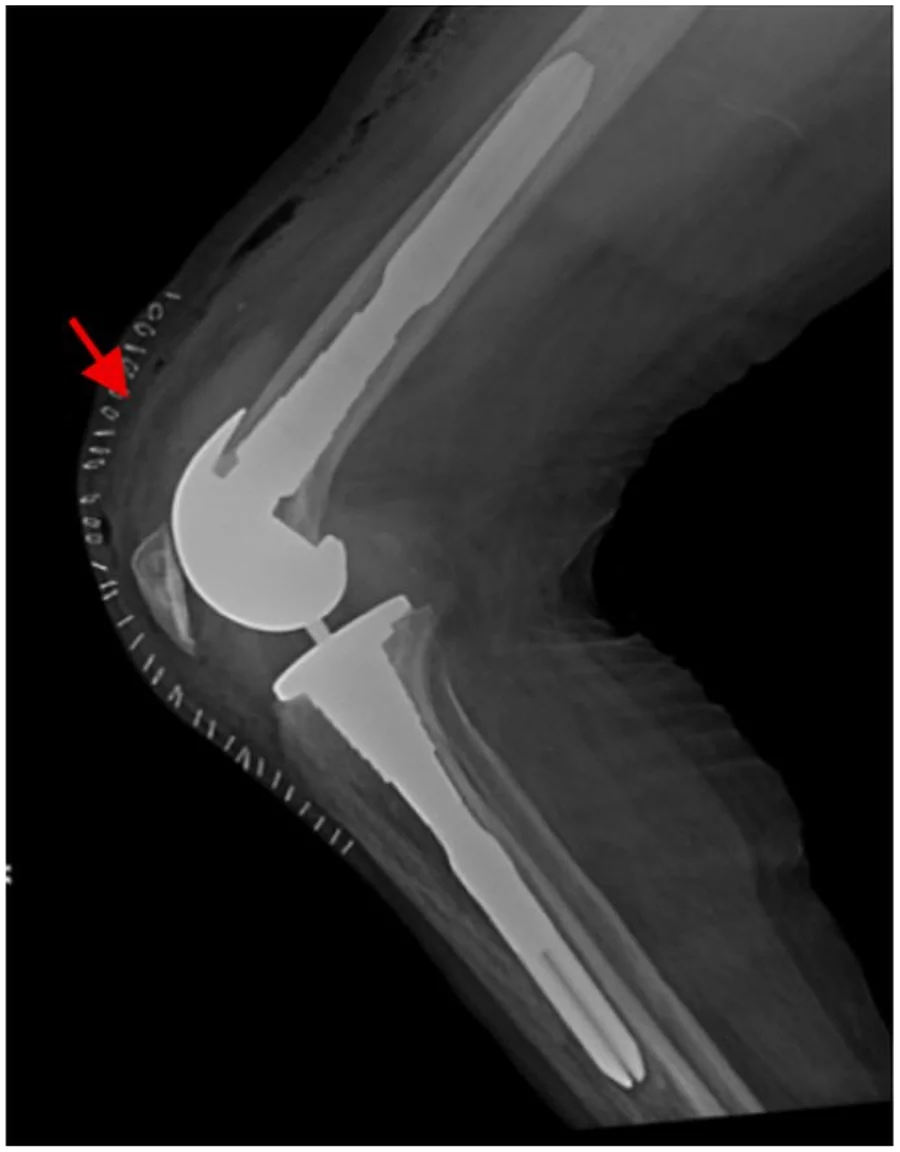
Lateral radiograph of the right knee obtained on 20 November 2025, after second-stage reimplantation of the right knee with revision components. There are no signs of acute hardware-related complication or loosening. Postoperative changes and surgical clips are noted, as well as a large joint effusion (red arrow).

He was reviewed at three-monthly intervals thereafter. At the first review after reimplantation, on February 19, 2026, he was clinically well, with a CRP of 14.15 mg/L attributed to postoperative inflammation. At the most recent review on May 11, 2026, approximately six months after reimplantation and 13 months after presentation, he had no complaints and was ambulating without an assistive device. The wound was healed with no sinus or discharge, range of motion was 0°–120°, and quadriceps strength and patellar tracking were good. CRP had fallen to 3.2 mg/L. Radiographs showed no loosening or hardware-related complication (Figure 6), and a gallium scan was negative. There has been no recurrence of infection, and no complication was recorded at any point in the course, including no wound problem, no adverse reaction to the antimicrobial regimen, and no hardware-related complication. He remains on three-monthly review with CRP, erythrocyte sedimentation rate, and bilateral knee radiographs, and continues physiotherapy. No validated patient-reported outcome instrument was administered during the episode, and functional outcome is therefore reported descriptively.

**Figure 6:**
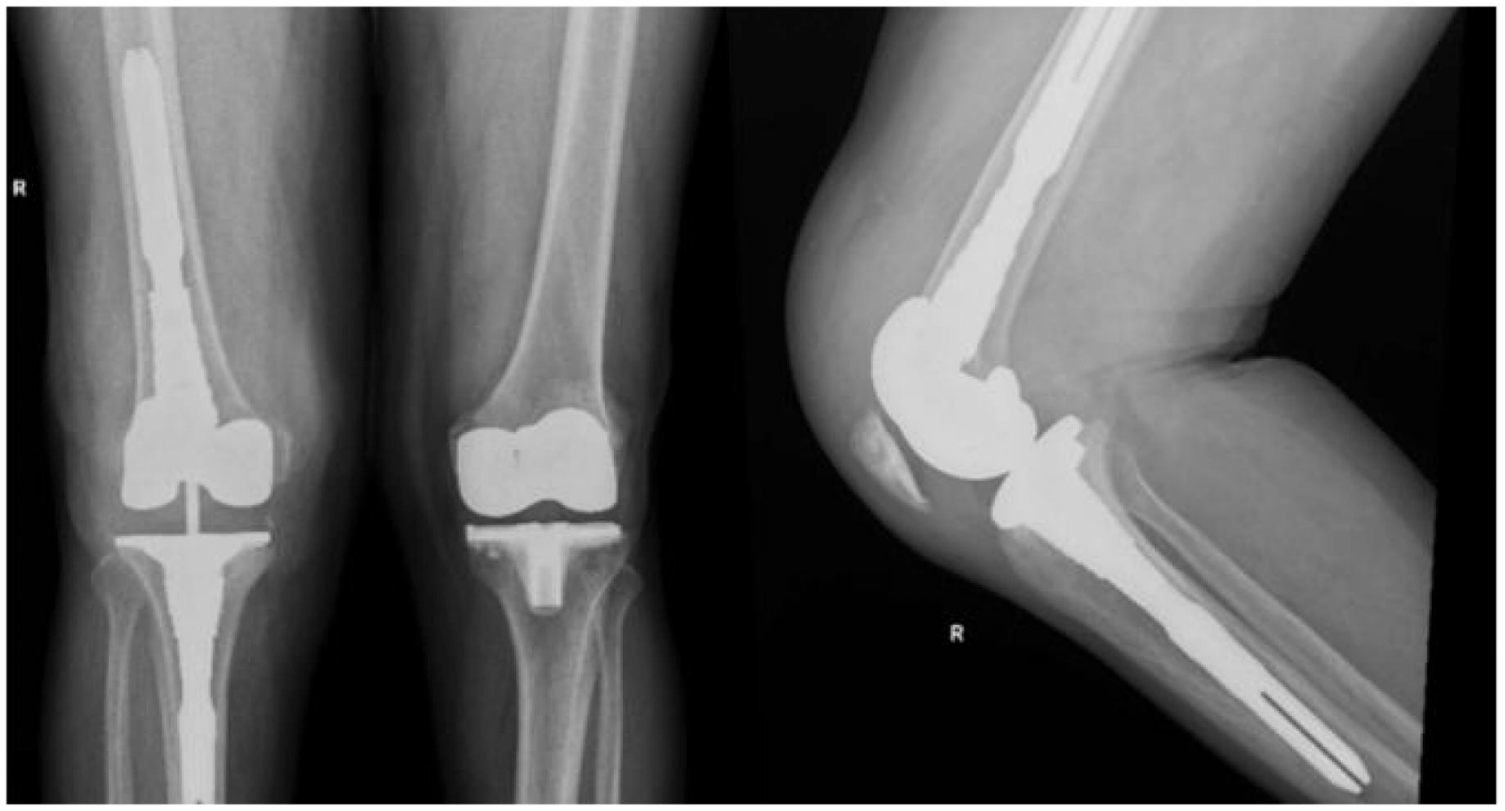
Radiographs at the most recent follow-up on 11 May 2026, approximately six months after second-stage reimplantation of the right knee with revision components. Left, anteroposterior view of both knees; right, lateral view of the right knee. There are no signs of acute hardware-related complication or loosening.

### 2.2 Literature review

PubMed and Scopus were searched on 1 March 2026 using the following search string: (prosth* OR replacement OR arthroplasty) AND (knee OR joint))) AND brucell*.

All retrieved records were screened without restrictions on publication date or language. Records were screened by two authors independently, first by title and abstract and then by full text, with disagreements resolved by discussion with a third author. Duplicate records were removed in Zotero, and duplicate publications of the same case were identified by cross-checking patient age, sex, institution, country, and year of report. Prosthetic joint brucellosis was defined by one of the following criteria: (a) isolation of *Brucella spp*. from synovial fluid obtained from a prosthetic joint; or (b) clinical features consistent with PJI accompanied by either isolation of *Brucella spp*. from blood cultures or positive serological evidence, defined as a standard agglutination test (SAT) titre >1:160 or a fourfold rise in titre between acute and convalescent samples. We excluded septic arthritis of native joints, osteoarticular brucellosis without prosthetic involvement, narrative reviews without primary case data, and conference abstracts without extractable data.

The final search identified 40 reported cases of *Brucella* infection occurring after TKA (Table 3) (1, 3, 5–33). The search yielded 167 records. After removal of 50 duplicates, 117 records were screened by title and abstract, of which 83 were excluded. All 34 full-text articles assessed met the eligibility criteria. The selection process is summarized in Figure 7.

**Table 3:** Comprehensive summary of previous cases of *Brucella* prosthetic knee infections.

Case (Author, Year)	Age/Sex	Country/Travel	Occupation	Latency (Months)	SAT Titre	Radiographic Changes	Blood Culture/Joint Culture	Organism	Antibiotics Used/Length (Wks)	Surgical Management	Outcome
Agarwal et al. (1991) (32)	24 F	Saudi/No	NR	2	1:2,560	No loosening	No/Yes	*B. melitensis*	Rif/TMP-SMX (76)	Medical only	Pain free
Orti et al. (1997) (33)	60 M	Spain/No	Goat worker	14	1:160	No loosening	No/Yes	*B. melitensis*	Strep/Doxy/Rif (6)	Medical only	Symptom free
Malizos et al. (1997) (31)	74 M	Greece/No	Shepherd	5	1:160	No loosening	Yes/Yes	*B. melitensis*	Strep/Doxy/TMP-SMX (28)	Medical only	Asymptomatic
Iglesias (1997)	71 F	Spain/No	NR	48	Pos	No loosening	NR/Yes	*Brucella spp.*	Doxy/Rif/Strep (18.5)	Medical only	Good
Weil et al. (2003) #2 (30)	64 M	Israel/No	Retired	30	1:1,600	Yes	NR/No	*B. melitensis*	Doxy/Rif (12)	2-Stage Revision	Pain free
Weil et al. (2003) #3 (30)	67 M	Israel/No	Retired	168	1:1,600	Yes	NR/Yes	*B. melitensis*	Doxy/Rif (12)	2-Stage Revision	Pain free
Marchese et al. (2006) (22)	74 M	Italy/No	NR	108	NR	Yes	NR/Yes	*B. melitensis*	Strep/Doxy/Rif/Levo (36)	Implant removal	Good
Marbach et al. (2007) (29)	67 NR	Sicily/Yes	NR	48	NR	Yes	NR/NR	*Brucella spp.*	Doxy/Rif (12)	2-Stage Revision	Good ROM
Tassinari et al. (2008) (27)	68 M	Italy/No	NR	24	1:800	No loosening	NR/Yes	*B. melitensis*	Doxy/Rif (8)	Medical only	Pain resolved
Atay (2008)	72 M	Turkey/No	NR	48	Pos	No loosening	No/Yes	*B. melitensis*	Doxy/Rif (6)	DAIR (Arthro)	Good
Dauty et al. (2009) (25)	65 F	Portugal/Yes	NR	NR	NR	Yes	NR/NR	*B. melitensis*	Doxy/Rif (12)	2-Stage Revision	Pain free
Erdogan et al. (2010) (28)	63 F	Turkey/No	NR	24	1:160	No loosening	NR/NR	*B. melitensis*	Doxy/Rif (20)	Medical only	Pain free
Wunschel et al. (2011) (21)	60 M	Turkey/No	NR	60	Pos	Yes	NR/NR	*Brucella spp.*	Doxy/Rif (24)	1-Stage Revision	Good
Oner et al. (2012) (19)	65 F	Turkey/No	NR	96	Pos	Yes	NR/Yes	*B. melitensis*	Doxy/Rif (16)	2-Stage Revision	Good
Karaaslan et al. (2014) (20)	62 M	Turkey/No	NR	24	Pos	Yes	NR/Yes	*B. melitensis*	Doxy/Rif (12)	2-Stage Revision	Good
Lewis et al. (2016) (26)	51 M	Thailand/Yes	Director	60	>1:2,560	No loosening	Yes/Yes	*B. melitensis*	Doxy/Rif/Gent (24)	Medical only	Well
Klassov et al. (2016) (18)	79 M	Arg-Isr/Yes	NR	144/48	IgG 1:640	Yes	NR/Yes	*B. melitensis*	Gen/Doxy/Rif/Bact (64+)	2-Stage Revision	Relapse noted
Jabalameli et al. (2016) (12)	68 F	Iran/No	NR	12	NR	Yes	No/Yes	*B. melitensis*	Rif/Doxy (24)	2-Stage Revision	Good
Flury et al. (2017) (6)	75 M	Turkey/Yes	NR	144	1:240	Yes	No/Yes	B. melitensis	Doxy/Gent/Rif/Pen (24)	2-Stage Revision	Good ROM
Sazegari et al. (2017) (15)	78 M	Iran/No	NR	60	Pos	Yes	NR/NR	*Brucella spp.*	Rif/Doxy (8)	2-Stage Revision	Successful
Mortazavi et al. (2017) (3)	28 M	Iran/No	NR	48	1:1,280	Yes	No/Yes	*B. melitensis*	Doxy/Rif/Bact/Cipro (100+)	2-Stage Revision	Excellent
Turvey (2017) (8)	65 F	Canada/No	Artist	132	<1:40 (Neg)	Yes	NR/Yes	*B. suis*	Doxy/Rif/Gent (12)	2-Stage Revision	Eradicated
Maalouf (2018)	63 F	Lebanon/No	NR	24	1:5,120	Yes	NR/NR	*Brucella spp.*	Gen/Rif/Doxy (16)	2-Stage Revision	Good
Balkhair et al. (2019) (17)	57 F	Oman/No	NR	120	Pos	No loosening	Yes/Yes	*B. melitensis*	Doxy/Rif/Gent (24)	Medical only	Pain free
Hamdi et al. (2019) (16)	73 F	Saudi/No	NR	144	NR	Yes	NR/No	*Brucella spp.*	Doxy/Rif/Strep (24)	2-Stage Revision	Fine
Jabalameli et al. (2021) (13)	63 F	Iran/No	Housewife	24	Pos	Yes	No/No	*Brucella spp.*	Rif/Doxy (24)	2-Stage Revision	Asymptomatic
Shokouhi et al. (2022) (14)	62 F	Iran/No	Teacher	60	1:160	No loosening	Yes/Yes	*B. melitensis*	Doxy/Rif/Gent (24)	Medical only	Cured
Athanasiou et al. (2022) (5)	78 M	Greece/No	Farmer	84	1:5,120	Yes	No/Yes	*B. melitensis*	Doxy/Gent/Rif (16)	2-Stage Revision	Good
Kuyubasi et al. (2022) (7)	70 F	Turkey/No	Agricul.	60	1:80–>1:640	Yes	No/Yes	*Brucella spp.*	Rif/Doxy/Ceft/Bact (12–16)	2-Stage Revision	Good
Köken (2023) (11)	58 F	Turkey/No	NR	72	1:320	Yes	No/No	*B. melitensis*	Rif/Doxy (24)	2-Stage Revision	Pain free
Stumpner et al. (2023) (1)	68 F	Afghan/Yes	Livestock	60	1/320	Yes	NR/Yes	*B. melitensis*	Doxy/Rif/Gent (12)	2-Stage Revision	Pain free
Chalbaoui et al. (2024) (24)	71 F	Tunisia/No	NR	48	1:80	Yes (Loosening)	No/NR	*Brucella spp.*	Doxy/Rif/Bact (30)	2-Stage Revision	Favorable
Abul et al. (2025) #1 (10)	Late 60s F	Turkey/No	NR	60	1:320–1:640	No	NR/No	*B. melitensis*	Doxy/Rif/Gent (12)	2-Stage Revision	Pain-free
Abul et al. (2025) #2 (10)	Mid-60 M	Turkey/No	NR	96	NR	Yes	NR/Yes	*B. melitensis*	Doxy/Rif (12)	1-Stage Revision	Success
Abul et al. (2025) #3 (10)	Early-60 M	Turkey/No	NR	72	Pos	No loosening	NR/NR	*B. melitensis*	Doxy/Rif (12)	Medical only	Success
Wang et al. (2025) (4 Knee cases) (23)	55–79 (Mean: 65.6)/M & F	China/No	Herdsmen	23.0 ± 12.5 (Mean)	≥1:160	Loosening (Detected in all)	NR/Positive (87.5% tissue; 100% PCR)	*Brucella spp.*	Doxy/Rif (12–24)	One-stage revision arthroplasty	Successful eradication; Mean RO
Fu et al. (2026) (9)	63 F	China/No	NR	13	Pos	No loosening	No/No	*Brucella spp.*	Doxy/Rif (12)	Medical only	Sinus healed
Present Case	67 M	Saudi/No	Farmer (sheep)	∼48	>1:160	Yes (Loosening)	No/Yes	*Brucella spp.*	Doxy/Rif (24 Total)	2-Stage Revision	Reimplanted 19 Nov 2025; pain free, ROM 0–120°, no recurrence at 6 mo

NR, not reported; SAT, serum agglutination test; Doxy, doxycycline; Rif, rifampicin; Strep, streptomycin; Gent, gentamicin; Bact, trimethoprim-sulfamethoxazole; Cipro, ciprofloxacin; Pen, penicillin; Levo, levofloxacin; Ceft, ceftriaxone; DAIR, debridement, antibiotics, and implant retention; ROM, range of motion.

**Figure 7:**
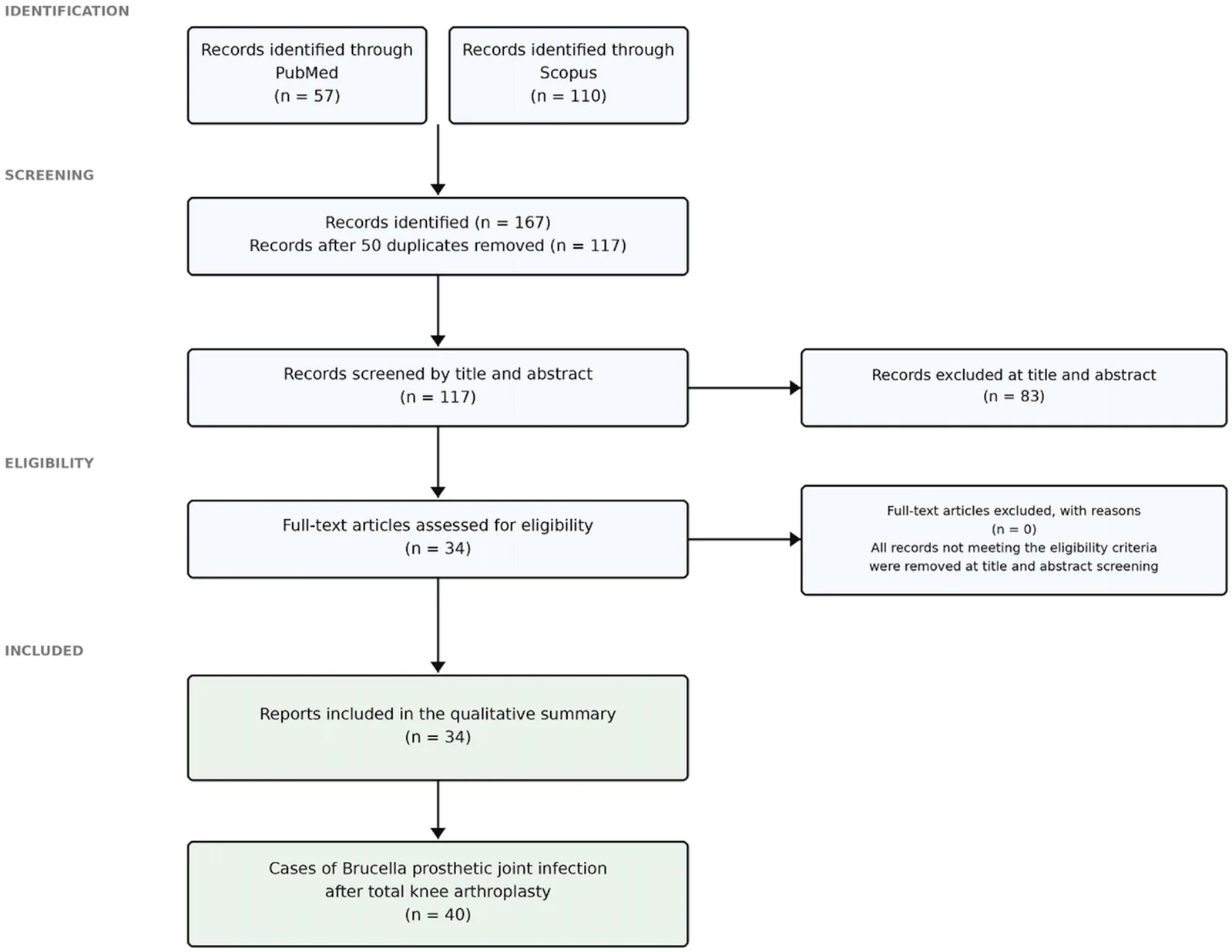
Flow diagram of the literature search and case selection. PubMed and Scopus were searched on 1 March 2026, yielding 167 records. After removal of 50 duplicates, 117 records were screened by title and abstract and 83 were excluded. All 34 full-text articles assessed met the eligibility criteria and were included, yielding 40 cases of *Brucella* prosthetic joint infection after total knee arthroplasty.

## 3 Discussion

The presentation of *Brucella* PJI in this 67-year-old male highlights several classic diagnostic and therapeutic challenges associated with this rare zoonotic complication. While the majority of PJIs are caused by Staphylococcus aureus or coagulase-negative staphylococci, *Brucella* species must be considered in the differential diagnosis of painful arthroplasty, particularly in patients from endemic regions or with relevant exposure histories (24). Exposure data were, however, among the least consistently reported variables in the cases reviewed here. Occupation was stated in only 12 of the 37 previously reported cases summarized in Table 3, dietary exposure was not reported in an extractable form, and travel was recorded only alongside country of residence, which limits any attempt to describe the route of acquisition across published cases.

A defining feature of this case is the latency period of approximately 48 months (4 years) from the index surgery to symptom onset. This aligns precisely with the median duration of 3.9–5.4 years reported across multiple reviews (6, 34). The indolent course, characterized by progressive pain and swelling without systemic symptoms like fever, is also typical; sources indicate that systemic manifestations occur in only 25%–38% of *Brucella* PJI cases. This absence of “undulant fever” often leads to misdiagnosis, frequently being mistaken for aseptic loosening (23).

The diagnosis in this case was confirmed through ultrasound-guided joint aspiration and intraoperative cultures. While synovial fluid culture positivity in *Brucella* PJI is relatively high (∼75%), the organism is fastidious and slow-growing, often requiring prolonged incubation for up to 21 days (34). This case's use of bone and gallium scintigraphy supported the impression of an infected prosthesis, and nuclear imaging is sensitive for detecting the hyperemia and focal uptake associated with periprosthetic inflammatory processes (1). Neither modality is specific. Both may remain positive in aseptic loosening and in the chronic inflammatory change that surrounds any implant, and neither identifies a causative organism, so a positive study in a painful arthroplasty should prompt sampling rather than substitute for it. In this patient the scans were performed after *Brucella* had already been recovered from the synovial fluid aspirate on 19 April 2025, and their contribution was to define the extent of periprosthetic involvement rather than to establish the diagnosis. The significantly elevated CRP (119.08 mg/L) in this patient is a common finding, though its absence does not rule out the diagnosis, as seen in Chalbaoui et al., where the CRP was only 34 mg/L (24).

The management of *Brucella* PJI is dictated primarily by the mechanical stability of the implant. Recent cases, such as Fu et al., demonstrate that even with a sinus tract, medical therapy alone can be successful if the components are radiographically stable (9).

In this case, radiographic loosening of the tibial component was identified, which mandates surgical intervention. The patient underwent a two-stage revision, which remains the traditional gold standard for chronic PJI with instability. However, very recent reports from Wang et al. and Abul et al. suggest that one-stage revision is a feasible and effective alternative in select *Brucella* cases due to the pathogen's lower adhesion to metal compared to other bacteria (10, 23). The choice of a two-stage approach here provided a controlled environment to ensure infection eradication before reimplantation. In this patient the decision was reinforced by microbiology rather than by preference. *Brucella* was still recovered from the intraoperative cultures taken at the first stage despite a completed three-month course of doxycycline and rifampin, which indicated that the organism persisted in a biofilm on the components and that retention would have been inadequate. Second-stage reimplantation was performed on November 19, 2025, once the full antibiotic course was complete and inflammatory markers had normalized, and all intraoperative cultures at that stage were negative.

The treatment utilized doxycycline and rifampin, which is the most widely recommended dual-regimen for both systemic brucellosis and PJI. The patient's completion of a three-month pre-operative course and planned three-month post-operative course (6 months total) is consistent with the median treatment duration found in literature. This prolonged therapy is justified by *Brucella's* ability to persist intracellularly within phagocytic cells, which often necessitates treatment for 12 weeks to 6 months to prevent relapse (9). The published experience is nonetheless heterogeneous. Across the 37 previously reported cases summarized in Table 3, treatment duration ranges from 6 to more than 100 weeks, 18 used doxycycline with rifampin alone and 15 used a regimen containing an aminoglycoside, and no report compares outcomes between them, so the optimal agent combination and duration in prosthetic joint infection remain unresolved.

The presentation in this patient carried no feature that would ordinarily raise suspicion of infection. He was afebrile throughout, had no sinus tract or wound complication, and the radiograph showed a focal lucency at the tibial component consistent with aseptic loosening. Gram stain was negative on the first two days, and the organism was recovered only after seven days of incubation. The synovial fluid analysis was itself discordant. Under the 2018 International Consensus Meeting criteria, the leukocyte count of 10,000 cells/µL exceeded the 3,000 cells/µL threshold, but the neutrophil proportion of 69% fell below the 80% cut-off, so the fluid analysis alone would not have secured the diagnosis (36). In endemic regions this combination argues for prolonged culture incubation and a low threshold for *Brucella* serology in any late prosthetic joint failure, even when the clinical picture appears aseptic.

*Brucella* is among the most frequently reported causes of laboratory-acquired infection (21). Transmission is by the aerosol route, the infecting dose is low, and the risk is greatest during open-bench manipulation of growth, including subculture, biochemical testing, and preparation of suspensions for identification. The hazard is compounded by the same features that make the organism difficult to diagnose. In the present case the Gram stain was negative on days 1 and 2 at both sampling episodes and Gram-negative coccobacilli appeared only after seven days of incubation, so the plates were handled before brucellosis was recognized. Where brucellosis is in the differential, the clinical team should record this on the request form and communicate it directly to the laboratory, so that manipulation of any suspicious growth is confined to a biological safety cabinet and referred to containment level 3 handling, and so that staff who have already worked with the plates can be identified for risk assessment and serological follow-up. This is of particular importance in the prosthetic joint setting, where the specimen is submitted as a routine periprosthetic sample and brucellosis is rarely the working diagnosis. The biosafety response in the present case, including what the microbiology laboratory was told and whether any staff exposure occurred or post-exposure follow-up was undertaken, was not recorded, and this is a limitation of the report.

This report has several limitations. It describes a single patient, and the findings cannot be generalized to the wider population with *Brucella* prosthetic joint infection. The exposure history relies on patient recall; no environmental or veterinary source investigation was undertaken, and household screening was not performed. Microbiological characterization was limited: the diagnosis rested on the growth of Gram-negative coccobacilli together with a positive Rose Bengal test and a standard tube agglutination titre above 1:160, but species-level identification, molecular confirmation, and antimicrobial susceptibility testing were not performed, so the infecting species and its susceptibility profile remain unknown. Molecular assays were available at our institution but were not requested, since *Brucella* had already been recovered in culture. In retrospect this was a missed opportunity, as *Brucella*-specific nucleic acid amplification would have provided species-level identification and a shorter interval to definitive identification, and we would now regard it, together with susceptibility testing on the isolate, as part of the standard work-up whenever brucellar prosthetic joint infection is suspected. Follow-up extends to approximately six months after second-stage reimplantation and 13 months from presentation, which is shorter than the two-year interval that international consensus regards as the minimum for reporting outcome after periprosthetic joint infection surgery (38); late relapse cannot be excluded, particularly given the recognized propensity of *Brucella* for recurrence. Functional outcome was recorded clinically rather than with a validated patient-reported outcome instrument. The spacer was loaded with vancomycin, which has no activity against *Brucella*, and a heat-stable agent with anti-*Brucella* activity such as gentamicin or streptomycin could reasonably have been used instead; rifampin is generally avoided in polymethylmethacrylate because it interferes with polymerization and degrades the mechanical properties of the cement (37). Finally, the optimal surgical strategy for *Brucella* prosthetic joint infection is not established. The evidence base consists of case reports and small series, and debridement with implant retention, one-stage exchange, and two-stage exchange have all been reported with success, so the two-stage approach used here reflects institutional practice and the extent of component loosening rather than comparative evidence.

Future research should focus on establishing standardized diagnostic and management algorithms for *Brucella* PJI. Multicenter registries and pooled analyses are needed to better define optimal antimicrobial regimens, treatment duration, and the role of one-stage vs. two-stage revision in relation to implant stability and host factors. The utility of advanced molecular diagnostics (e.g., PCR-based assays) and novel imaging modalities for earlier detection also warrants further investigation. Additionally, studies evaluating strategies for occupational safety and laboratory biosafety in suspected *Brucella* cases are essential to reduce nosocomial transmission.

## 4 Conclusion

*Brucella* PJI is a rare but clinically significant complication of total knee arthroplasty, characterized by delayed presentation and diagnostic complexity. Timely recognition, appropriate microbiological confirmation, and a combined medical–surgical approach tailored to implant stability are critical for infection eradication and favorable functional outcomes. In this patient a completed two-stage exchange, combined with two three-month courses of doxycycline and rifampin, achieved a pain-free knee with a full range of motion and no evidence of recurrence six months after reimplantation.

## Data Availability

The original contributions presented in the study are included in the article/Supplementary Material, further inquiries can be directed to the corresponding author.
